# *Potentilla reptans* L. postconditioning protects reperfusion injury via the RISK/SAFE pathways in an isolated rat heart

**DOI:** 10.1186/s12906-021-03456-2

**Published:** 2021-11-26

**Authors:** Ayesheh Enayati, Aref Salehi, Mostafa Alilou, Hermann Stuppner, Mirali Polshekan, Maryam Rajaei, Mona Pourabouk, Ali Jabbari, Zohreh Mazaheri, Narguess Yassa, Hamid Reza Moheimani, Vahid Khori

**Affiliations:** 1 Ischemic Disorders Research Center, Golestan University of Medical Sciences, P.O.BOX. 4934174515, Gorgan, Iran; 2 Institute of Pharmacy/Pharmacognosy, Center for Molecular Biosciences Innsbruck, University of Innsbruck, Innrain 80/82, 6020 Innsbruck, Austria; 3 Research Clinical Development unit (CRDU) 5 Azar Hospital, Golestan University of Medical Sciences, Gorgan, Iran; 4 Department of Pharmacognosy, Faculty of Pharmacy and Medicinal Plants Research Center, Tehran University of Medical Sciences, Tehran, Iran

**Keywords:** *Potentilla reptans* root, Postconditioning, Anti-apoptotic, Antioxidant, NO, GSK-3β, SGK1, Ischemia/reperfusion

## Abstract

**Background:**

Our previous study indicated that *Potentilla reptans* root has a preconditioning effect by its antioxidant and anti-apoptotic effects in an isolated rat heart ischemia/reperfusion (IR) model. In the present study, we investigated the post-conditioning cardio-protective effects of *Potentilla reptans* and its active substances.

**Methods:**

The ethyl acetate fraction of *P. reptans* root (Et) was subjected to an IR model under 30 min of ischemia and 100 min of reperfusion. To investigate the postconditioning effect, Et was perfused for 15 min at the early phase of reperfusion. RISK/SAFE pathway inhibitors, 5HD and L-NAME, were applied individually 10 min before the ischemia, either alone or in combination with Et during the early reperfusion phase. The hemodynamic factors and ventricular arrhythmia were calculated during the reperfusion. Oxidative stress, apoptosis markers, GSK-3β and SGK1 proteins were assessed at the end of experiments.

**Results:**

Et postconditioning (Etpost) significantly reduced the infarct size, arrhythmia score, ventricular fibrillation incidence, and enhanced the hemodynamic parameters by decreasing the MDA level and increasing expression of *Nrf2*, SOD and CAT activities. Meanwhile, Etpost increased the BCl-2/BAX ratio and decreased Caspase-3 expression. The cardioprotective effect of Etpost was abrogated by L-NAME, Wortmannin (a PI3K/Akt inhibitor), and AG490 (a JAK/STAT3 inhibitor). Finally, Etpost reduced the expression of GSK-3β and SGK1 proteins pertaining to the IR group.

**Conclusion:**

*P. reptans* reveals the post-conditioning effects via the *Nrf2* pathway, NO release, and the RISK/SAFE pathway. Also, Etpost decreased apoptotic indexes by inhibiting GSK-3β and SGK1 expressions. Hence, our data suggest that Etpost can be a suitable natural candidate to protect cardiomyocytes during reperfusion injury.

**Supplementary Information:**

The online version contains supplementary material available at 10.1186/s12906-021-03456-2.

## Background

Cardiac Ischemic postconditioning (IPOST) refers to the brief periods of ischemia/reperfusion (IR) cycles that occur at the early phase of reperfusion [1, 2]. Reactive oxygen species (ROS) play an essential role in ischemic postconditioning via the intervention of redox signaling using either ROS scavengers or ROS generators during the early myocardial reperfusion [1]. The massive production of ROS and the occurrence of myocardial injury lead to the induction of apoptosis and necrosis in cardiomyocytes during IR damage [3]. Postconditioning triggers other cardioprotective signaling cascades, such as the activation of NO, mitoKATP channel, RISK; PI3K/Akt, ERK1/2, SAFE; JAK/STAT3 signaling pathways, PKC, protein tyrosine kinase, and mitogen-activated protein kinase [2, 4]. Additionally, there are a large number of strategies to protect cardiac reperfusion induced injury related to those mechanisms, however, the clinical approaches are still not satisfied and safe. The previous studies showed that medicinal plants such as *Potentilla* species and their active ingredients including flavan-3-ols can suppress ROS and incite the release of NO through the down/upregulation of several signaling cascades involved during reperfusion injury [5–7]. Therefore, it could be valuable, finding new natural pharmacological agents that protect cardiac against IR injury by modifying involved mechanisms.

*Potentilla reptans* L. (Rosaceae) is a medicinal herbaceous, which known to have cardioprotective properties [5–7]. Recent updates, the chemical constituents of *P. reptans* root has been assessed for its phytochemical studies in tannins and triterpenoids [5, 6, 8]. The previous studies showed that *P. reptans* revealed various biological and pharmalogical activities including antioxidant, anti-inflammatory, cytotoxic and enzyme inhibitory potential, tumor therapeutic potential, and antiulcer activity [5, 9–11]. In line with above findings, our previous study reported antioxidant activity of various fractions of *P. reptans* root [6]. In addition, we presented cardioprotective functions of *P. reptans* root by cardiac ischemic preconditioning effect in an isolated rat heart ischemia/reperfusion (IR) model. It protected IR injury due to NO release, ROS inhibition, activation of Nrf2 pathway, and reduction of apoptosis in rat myocardial [7]. To the best of our knowledge, the ischemic postconditioning effect of *Potentilla reptans* root have not been investigated. Thus, in the current study, we aimed to evaluate the cardioprotective effect of ethyl acetate fraction of *Potentilla reptans* root based on its underlying mechanisms in NO, *Nrf2*, mitoKATP, RISK/SAFE signaling pathways, GSK-3β, SGK1, and anti-apoptotic index pertaining to the recorded evidences of pharmacological in the rat heart IR injury.

## Methods

### Plant collection and identification

The roots of *Potentilla reptans* L. were collected (in June 2015) from Tangrah village located in the North region of Iran (37° 23′ 50″ N;55° 46′ 55″ E). Plant specimens were authenticated and kept by a botanist, Dr. Farideh Attar at the Central Herbarium, Tehran University, Iran with a voucher specimen (45815-TUH). All plant protocols were performed in accordance with the Herbal Medicinal Product Committee (HMPC) (Ref. EMEA/HMPC/246816/2005) guideline and Research Ethics Committees of Golestan University of Medical Sciences, Golestan, Iran [6, 7].

### Preparation of plant extract

The extraction process of *Potentilla reptans* root was performed as previously described [7]. The *P. reptans* ethyl acetate fraction was dissolved and sonicated in water.

### Animal care

Male Wistar rats (220–260 g, 7-8 weeks old) were obtained from the animal center, Golestan University of Medicinal Sciences, Gorgan, Iran. Rats were maintained in cage (3-4 rats/cage) under standard conditions with a 12-h light-dark cycle, an ambient temperature of 20–23 °C, and 40–50% humidity. The animals had free access to normal food and water. Animal procedures were performed according to the guidelines of the National Institutes of Health (NIH Publication No. 85-23, revised 2011) and ARRIVE guidelines [12–14]. All experimental protocol was approved by the Animal Ethics Committee (IR.GOUMS.REC.1399.021) of Golestan University of Medical Sciences, Golestan, Iran [6, 7].

### Drugs

Evans blue, NaCl, NaHCO_3_, KCl, NaH_2_PO_4_, CaCl_2_, MgCl_2_, Glucose, and 2, 3, 5-triphenyl-tetrazolium chloride (TTC) were all purchased from Merck (Kenilworth, NJ). L-NAME (Nω-nitro-L-Arginine methyl ester-a nonspecific NOS inhibitor) was bought from Sigma-Aldrich (Billerica, MA). Wortmannin (Wort; the PI3k/Akt inhibitor), PD98059 (PD; the ERK1/2 inhibitor), Tyrphostin AG490 (the JAK/STAT3 inhibitor), and 5-hydroxy decanoate (a mitoKATP channel blocker) were purchased from “Santa Cruz Biotech, Dallas, Texas, USA”. The antioxidant determination Kits were purchased from ZellBio GmbH (Germany). The HPLC-grade solvents for column chromatography (CH_3_CN, MeOH, and acetone), were purchased from Sigma-Aldrich, Germany. All compounds were water-soluble.

### Isolated hearts

All surgical procedures used in the present study had been previously explained [2, 4, 6, 7]. Briefly, animals were anesthetized with a combination of 10% ketamine and 1% xylazine (100/10 mg/kg, IP) and then, heparin (200 IU/kg, IP) was injected as an anti-coagulation agent. After 1 min, surgery was performed when rats were completely anesthetized and did not show any toe pinch and corneal reflex. The exposed trachea was connected to a rodent ventilator (Model 683, Harvard Apparatus, Holliston, MA, USA) by a fit cannula. The aorta was cannulated and the hearts were rapidly excised and immersed in ice-cold Krebs-Henseleit buffer. Then, the hearts were implanted on Langendorff perfusion system at a constant pressure of 75 - 80 mmHg (depending the coronary flow) with an oxygenated (95% O_2_, 5% CO_2_, 37 °C) Krebs-Henseleit buffer (in mM: 118 NaCl, 25 NaHCO_3_, 4 KCl, 1.2 NaH_2_PO_4_, 1.9 CaCl_2_, 1.2 MgCl_2_, 11.1 Glucose, pH 7.35–7.45). The hearts were perfused with the Krebs-Henseleit buffer for 30 min. Next, a 30 min regional ischemia was performed in the left anterior descending (LAD) artery by occlusion using a silk string (6–0 mm), followed by 100 min (mins) of reperfusion.

### Experimental protocol

**Fig. 1:**
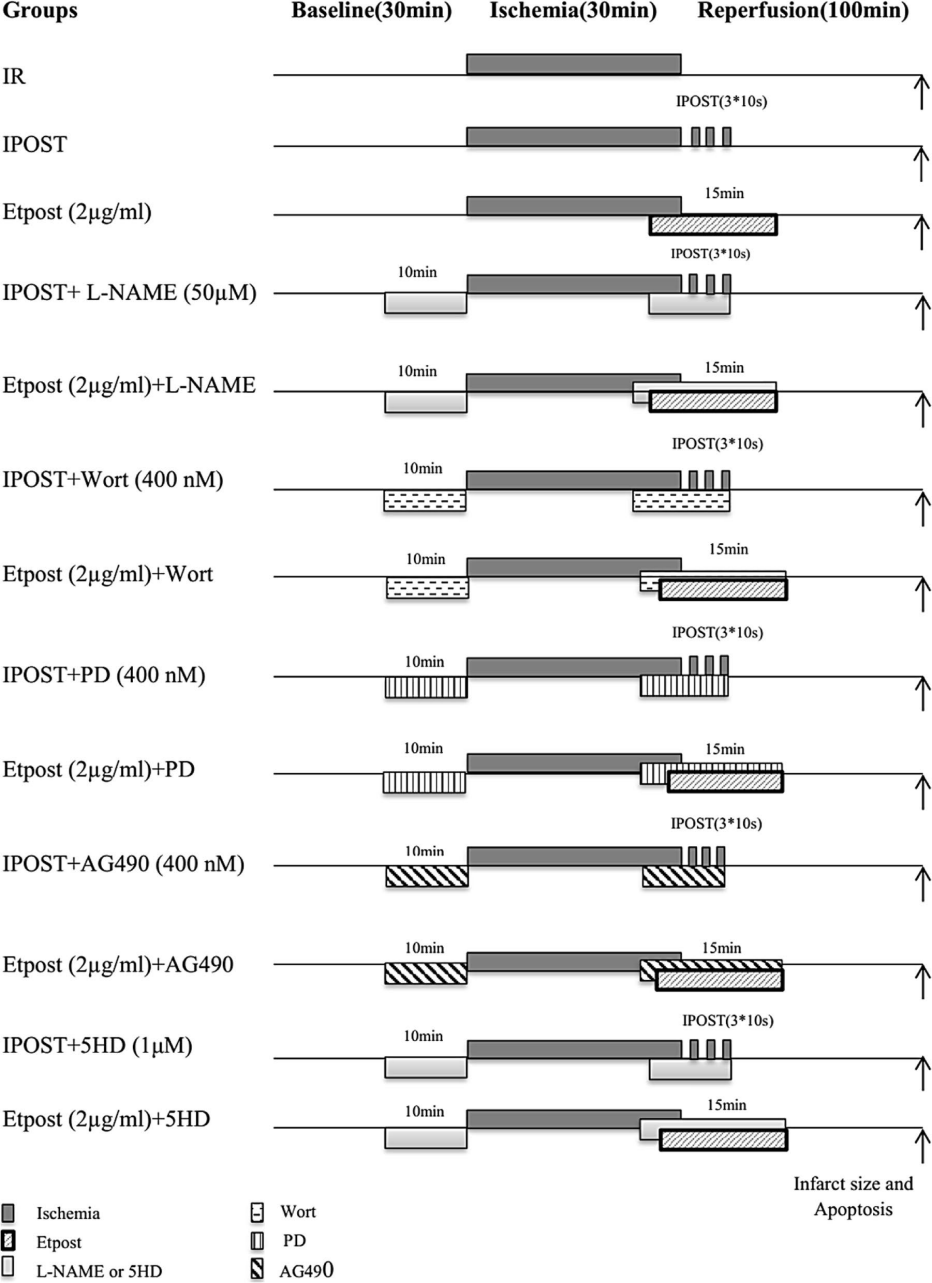
Experimental protocols. All experimental groups were first perfused for 30 mins on the Langendorff apparatus to allow the isolated hearts to stabilize. The hearts were then divided into different groups. All groups were subjected to 30 mins of regional ischemia followed by 100 mins of reperfusion. IR; Ischemia-reperfusion, IPOST; Ischemic postconditioning (3 × 10 s ischemia and reperfusion at the onset of reperfusion period), Etpost; ethyl acetate fraction from *Potentilla reptans* root (2 μg/ml) was applied at the onset of reperfusion, L-NAME; NO inhibitor (50 μM), Wort; Wortmannin a PI3K/AKT inhibitor (400 nM), PD; PD98059 (2′-Amino-3′-methoxyflavone) an ERK1/2 inhibitor (400 nM), AG; tyrphostin (AG490, 400 nM) a JAK/STAT3 inhibitor, HD; 5-hydroxy decanoate (5HD,1 μM) a mitoKATP channel blocker

Rats were randomly divided into 13 groups. The sample size was calculated 6 sample per group using G*Power software (α = 5%, effect size =0.63, power = 90%). The hearts were perfused and stabilized (baseline), then the hearts were subjected to 30 mins of global ischemia induced by the left anterior descending (LAD) artery occlusion using a silk string (6-0 mm), followed by 100 mins reperfusion. The following protocols were performed: 1: IR (rats that received no treatment); 2: ischemic postconditioning (IPOST), which was achieved by 3 episodes of 10 s ischemia and 10 s reperfusion at the onset of the reperfusion phase; 3: Etpost (2 μg/ml) was added in Krebs-Henseleit buffer and applied through the first 15 mins of the reperfusion phase; 4: Etpost (2 μg/ml) + L-NAME(50 μM); 5: Etpost(2 μg/ml) + Wort(400 nM); 6: Etpost(2 μg/ml) + PD(400 nM); 7: Etpost(2 μg/ml) + AG (tyrphostin AG490, 400 nM); 8: Etpost (2 μg/ml) + 5HD (1μΜ); 9: L-NAME(50 μM) + IPOST +IR; 10: Wort(400 nM) + IPOST+IR; 11: PD(400 nM) + IPOST+IR; 12: AG490(400 nM) + IPOST+IR; 13: 5HD(1 μM) + IPOST+IR. The inhibitors were applied individually, at 10 mins prior to the induction of global ischemia and in combination with Etpost during the first 15 mins of the reperfusion phase [2, 4] as shown in Fig. 1. Based on our previous study the effective concentration (EC_50_) of ethyl acetate fraction of *P. reptans* (Et) was determined at 2 μg/ml in a preconditioning mechanism. Thus, we used this Etpost at a concentration of 2 μg/ml [7].

### Hemodynamic parameters

A latex water-filled balloon was used to measure hemodynamic parameters, inserted into the left ventricle which was connected to a pressure transducer. The heart rate (HR), the left ventricular developed pressure (LVDP = LVSP − LVEDP), the left ventricular systolic pressure (LVSP), the rates of pressure development and fall (dP/dt max and dP/dt min), and the rate pressure product (RPP = LVDP × HR ÷ 1000) were monitored by the Power Lab software (Power Lab 8/30 AD Instruments, Australia) [2]. The coronary flow (CF) was measured at the end of the reperfusion period.

### Infarct size determination

To identify infarct size and area at risk, at the end of the reperfusion period, TTC staining (2, 3, 5-triphenyl-tetrazolium chloride) was performed as previously described [7]. The infarct size was determined using the computerized planimetry technique (Photoshop, ver. 7.0, Adobe system, San Jose, CA, USA). The infarct size (IS) was measured and is expressed as a percentage of the area at risk (IS/AAR%), while the total area at risk (AAR) was expressed as the percentage of the ratio of the left ventricle (AAR/LV%) [2, 7].

### Real time PCR analysis for Nrf2

The expression of *Nrf2* was investigated on the left ventricular myocardial tissue on the 7300 ABI Real-Time PCR system (Applied Biosystems, Foster City, California, USA). The primer sequence used in this study was the same as the primers applied in our previous research and are listed in Table 1. The target gene expression was normalized against the housekeeping *GAPDH*, and the relative expression was determined using the 2^−∆∆Ct^ method [7].

**Table 1:** Primer sequence design for Real Time PCR assessment

Accession number	Gene	Primer sequence	Product length (Bp)	Melt Temperature (°***C***)
XM_006234398.3	Nrf2	F 5′ GAAAACGACAGAAACCTCCATC 3′ R 5′ CTCCATCCTCCCGAACCT 3′	202	83
XM_017593963.1	GAPDH	F 5′ CAT ACT CAG CAC CAG CAT CAC C 3′ R 5′ AAG TTC AAC GGC ACA GTC AAG G 3’	121	79.65

### Assessment of oxidative stress

The frozen tissue was lysed and homogenized in lysis buffer and then centrifuged to precipitate the insoluble materials. The resulting supernatant was employed for the assay. The concentration of malondialdehyde (MDA), as well as the endogenous antioxidants, namely SOD and CAT, was assessed by the ELISA method using commercial kits (ZellBio GmbH, Germany) according to their manufacturer’s recommendations [7].

### Assessment of ventricular arrhythmias

Ventricular arrhythmias were evaluated in accordance with the Lambeth Conventions [7]. Three forms of ventricular arrhythmias were analyzed; the premature ventricular complex (PVC) and bigeminy, ventricular tachycardia (VT) and ventricular fibrillation (VF), as well as arrhythmia score [2, 4].

### Immunohistochemistry

The immunohistochemical analysis was performed, as previously described for the assessment of BAX, BCl-2, and Caspase-3 expression in cardiomyocytes resident in the LV [7].

### TUNEL staining

Total caspase 3 abundance in the rat heart sections was measured using the TUNEL assay (In-Situ Cell Death Detection Kit) according to the protocols introduced by previous studies [4, 7].

### Western blot analysis

Western blot analysis was performed on the LV sections to evaluate the expression of BAX (LS-C353915, LSBio, Seattle, US), BCl-2 (BS-4563R, Bioss Inc., Boston, US), Caspase3 (SC-56046, Santa Cruz Biotechnology, CA, US), GSK-3β (NBP1-47470, Novus Biologicals, Manama, US), SGK1 (SC-28338, Santa Cruz Biotechnology, CA, US), P-AKT (Biotin, orb501663, Biorbyt, UK), P-ERK1/2 (orb10606, Biorbyt, UK), P-STAT3 (orb704362, Biorbyt, UK) proteins as previously described in detail elsewhere [7]. GAPDH (ab181602, Abcam, Cambridge, US) was used as the loading control. The intensity of each protein band was semi-quantified by the Image J image analysis system.

### Statistical assessment

Data were expressed as means ± SEM and analyzed by Graph Pad-Prism 6 software (San Diego, CA) and SPSS 16. The differences between groups were evaluated by using a one-way analysis of variance and the post hoc Tukey test. Hemodynamic data within and between groups were performed with a two-way analysis of variance. Kruskal-Wallis and Fisher’s exact test was applied for analyzing arrhythmia scores and VF incidence, respectively. *P* values < 0.05 were the considered significant levels.

## Results

### Post-ischemic injury

**Fig. 2:**
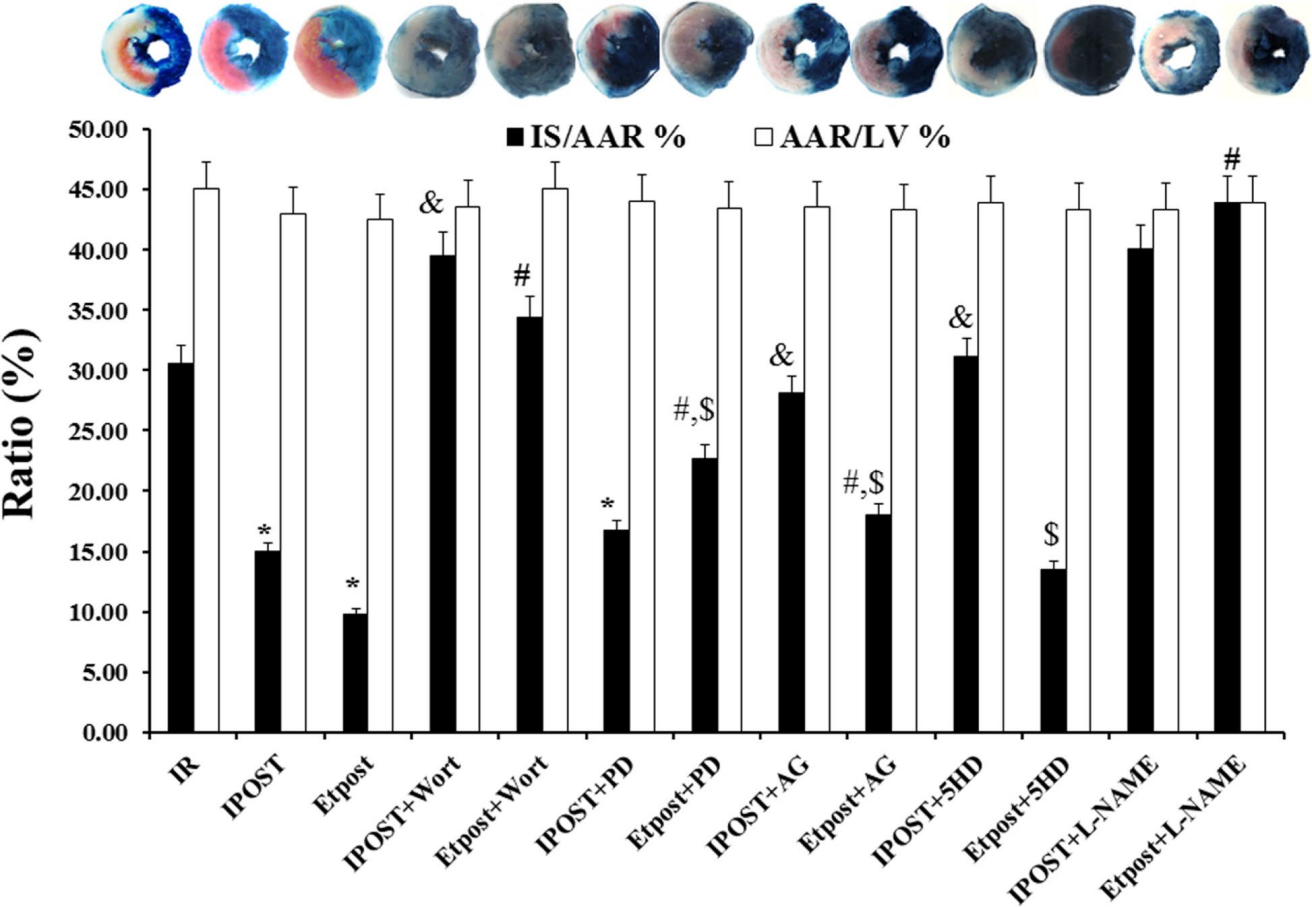
Cardioprotective effect of Etpost from *Potentilla reptans* root. Representative photographs of TTC (2,3,5-triphenyl-tetrazolium chloride) stained rat heart sections and statistical data of myocardial infarct size (IS/AAR%) and area at risk (AAR/LV%) in the isolated perfused rat hearts subjected to IR, IPOST, Etpost (2 μg/ml), IPOST + inhibitors, Etpost + inhibitors. Data are presented as means ± SEM and expressed in percentages. *P < 0.05 vs. IR; ^&^ *P* < 0.05 vs. IPOST; ^#^ *P* < 0.05 vs. Etpost (2 μg/ml); ^$^ *P* < 0.05 vs. IPOST+ inhibitor. (LV: Left ventricular, Etpost: ethyl acetate fraction of *P. reptans* root at postconditioning mechanism)

Etpost (2 μg/ml) significantly decreased infarct size (% IS/AAR = 9.79) compared with the IR (% IS/AAR = 30) group. The results revealed that Wort, PD, AG, 5HD, and L-NAME abolished the cardioprotective role of Etpost (9.79 ± 1.1) in the reduction of IS, but the effect of 5HD was not significant (13.49 ± 2.6) (*P* < 0.05, Fig. 2).

In the Etpost (2 μg/ml) group (6.1 ml/min), the coronary flow during the reperfusion phase was increased in comparison with the IR group. L-NAME (4.75 ml/min; 22%), Wort (≅ 50%), AG (≅ 50%) and 5HD (≅ 66%) abrogated the beneficial effect of Etpost (2 μg/ml) on the coronary flow during the reperfusion phase. PD (ERK inhibitor; 66%) increased the coronary flow when it was administered with Etpost (2 μg/ml) (*P* < 0.05, Table 2).

**Table 2:** Effect of Etpost (2 μg/ml) against the RISK/SAFE kinase inhibitors on functional hemodynamic parameters of the isolated rat heart during the reperfusion time

	% LVDP (mmHg)	% RPP (mmHg).(bpm)	% Heart Rate (bpm)	%Contractility Index (1/s)	% dP/dtmax (mmHg/s)	% dP/dtmin (mmHg/s)	Coronary Flow(ml/min)
IR (Con.)^@^	39.35 ± 15.9	35.42 ± 14.3	121.8 ± 20.4	39.75 ± 16.7	38.67 ± 15.7	34.49 ± 12.9	4.75 ± 0.25 [7]
IPOST (Con.)	79.93 ± 9.52^*^	73.30 ± 6.70^*^	91.62 ± 5.73^*^	67.76 ± 13.5^*^	72.91 ± 6.98^*^	73.26 ± 6.51^*^	7.56 ± 0.05^*^
Etpost (2 μg/ml)	78.81 ± 3.22^*^	67.24 ± 3.76^*^	85.62 ± 4.57^*^	77.69 ± 3.94^*^	75.36 ± 3.61^*^	65.85 ± 6.07^*^	6.10 ± 0.06^*^
Etpost (2 μg/ml) + Wort	66.09 ± 12.6^**#’$**^	67.89 ± 11.2^**$**^	101.38 ± 15.7^**#, $**^	72.64 ± 13.6^**$**^	69.62 ± 12.7^**$**^	60.19 ± 7.16^**$**^	3.28 ± 2.42^#^
Etpost (2 μg/ml) + PD	72.85 ± 12.4^**$**^	72.69 ± 13.3^**$**^	98.58 ± 5.03^**#**^	85.78 ± 8.93^**#, $**^	82.92 ± 15.6^**#, $**^	66.52 ± 12.7^**$**^	10.3 ± 1.11^#^
Etpost (2 μg/ml) + AG	56.37 ± 14.7^**#**^	47.10 ± 19.4^**#**^	70.26 ± 13.9^**#**^	62.13 ± 15.1^**#, $**^	52.53 ± 15.7^**#**^	46.43 ± 16.9^**#**^	3.05 ± 2.42^#, **$**^
Etpost (2 μg/ml) + HD	74.07 ± 2.86^**$**^	66.83 ± 2.91^**$**^	90.73 ± 7.57	123.9 ± 5.91^**#, $**^	76.13 ± 1.85^**$**^	67.38 ± 1.67^**$**^	2.84 ± 0.34^#^
IPOST + Wort	51.73 ± 9.77^**&**^	48.89 ± 9.34^**&**^	73.54 ± 15.92^**&**^	81.72 ± 11.5^**&**^	57.70 ± 8.94^**&**^	49.86 ± 7.95^**&**^	3.83 ± 1.42^&^
IPOST + PD	43.70 ± 9.97^**&**^	42.06 ± 11.9^**&**^	94.85 ± 16.5	40.64 ± 11.0^**&**^	44.97 ± 7.73^**&**^	37.76 ± 5.65^**&**^	5.10 ± 0.77^&^
IPOST + AG	49.39 ± 13.2^**&**^	46.71 ± 12.2^**&**^	78.87 ± 1.90^**&**^	99.48 ± 26.6^**&**^	54.59 ± 18.7^**&**^	47.38 ± 19.9^**&**^	0.75 ± 0.58^&^
IPOST + HD	61.62 ± 8.18^**&**^	57.83 ± 9.10^**&**^	90.66 ± 5.43	65.33 ± 15.1	57.47 ± 13.1^**&**^	56.95 ± 10.0^**&**^	3.00 ± 1.14^&^

*IR* Ischemic reperfusion, *IPOST* Ischemic postconditioning, *dp/dtmax* The steepest slope during the upstroke of the pressure, *dp/dt min* The steepest slope during the downstroke of the pressure, *LVDP* Left ventricular developed pressure (LV systolic pressure minus LV diastolic pressure), *RPP* Rate-Pressure Product, *Etpost* Ethyl acetate fraction of *Potentilla reptans* root in postconditioning mechanism, *Wort* Wortmannin (PI3K/Akt inhibitor), *PD* PD98059 (ERK inhibitor), *AG* AG490 a JAK/STAT3 inhibitor, *HD* 5HD a mitoKATP channel blocker. Data are presented as means ± SEM. *P < 0.05 vs. IR; ^&^ *P* < 0.05 vs. IPOST; ^#^ *P* < 0.05 vs. Etpost (2 μg/ml); ^$^ *P* < 0.05 vs. IPOST+inhibitor. @: [7]

### Real-time PCR analysis for Nrf2

**Fig. 3:**
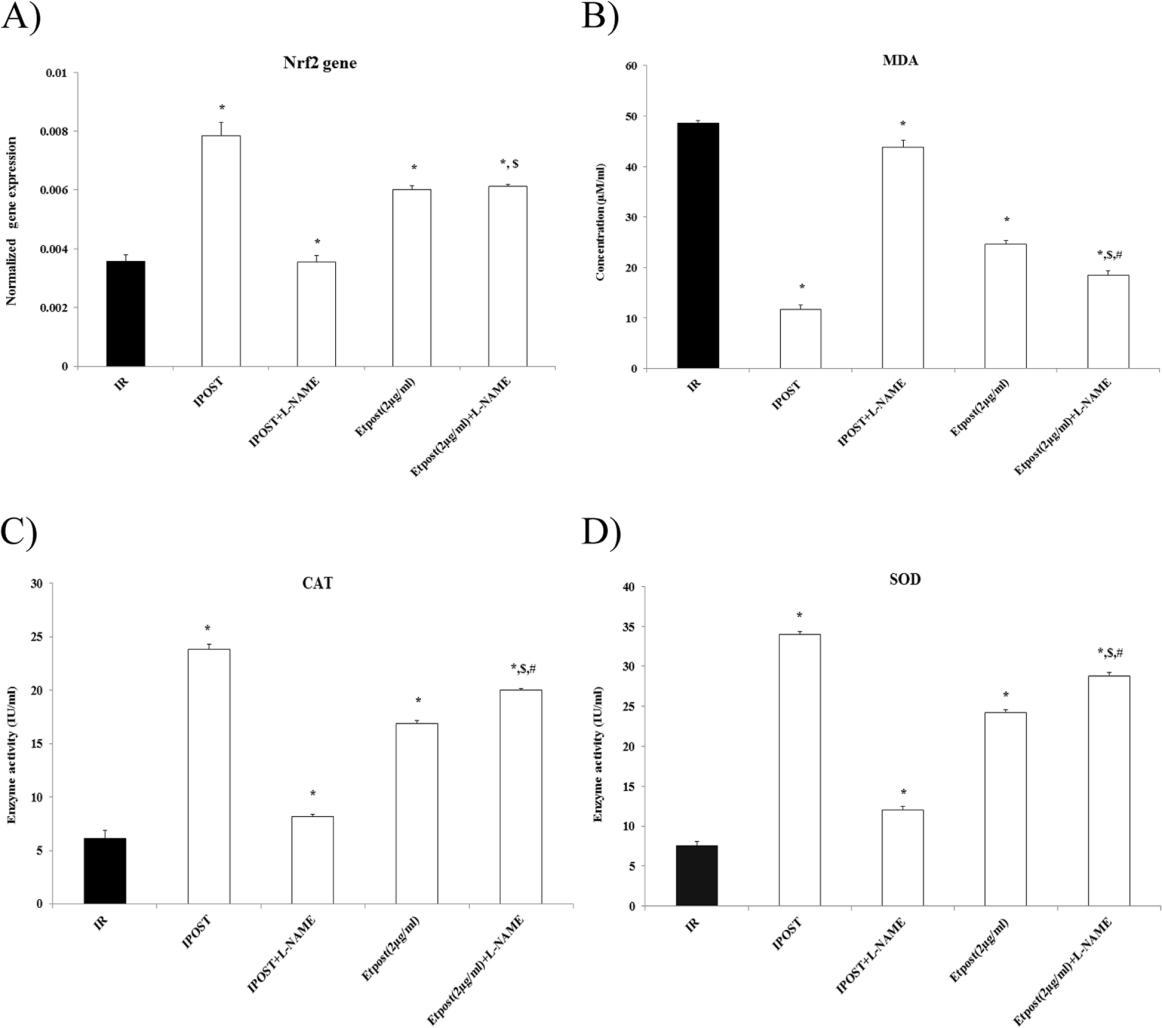
Antioxidant effect of Etpost (2 μg/ml) on the isolated rat heart tissue. Antioxidant effect of Etpost (2 μg/ml) on the isolated rat heart tissue. The effect of IR, L-NAME, Etpost (2 μg/ml), and Etpost (2 μg/ml) + L-NAME on oxidative stress markers. **A** The expression of *Nrf2* mRNA, **B** malondialdehyde (MDA), C) catalase (CAT), and D) superoxide dismutase (SOD). Data are presented as means ± SEM. ^*^P < 0.05 vs. IR, ^$^*P* < 0.05 vs. IPOST+L-NAME and ^#^*P* < 0.05 vs. Etpost (2 μg/ml)

The expression of Nrf2 genes significantly was increased by 50 and 33.34% in IPOST and Etpost (2 μg/ml) vs. IR group, respectively. Administration of L-NAME with Etpost did not change the *Nrf2* levels compared with Etpost group. While, in the Etpost (2 μg/ml) + L-NAME group, *Nrf2* levels were increased vs. IPOST+ L-NAME (Fig. 3A).

### Assessment of oxidative stress

Etpost (2 μg/ml) significantly decreased MDA levels (≅ 50%) against the IR group. Besides, L-NAME+Etpost (2 μg/ml) at the early reperfusion phase significantly decreased MDA levels as opposed to the Etpost (2 μg/ml) group (Fig. 3B). Etpost (2 μg/ml) on the other hand, strongly increased the SOD and CAT activities. Similar administration of L-NAME together with Etpost (2 μg/ml) increased their activities when compared with the Etpost (2 μg/ml) group only (Fig. 3C-D).

### Immunohistochemical assay

**Fig. 4:**
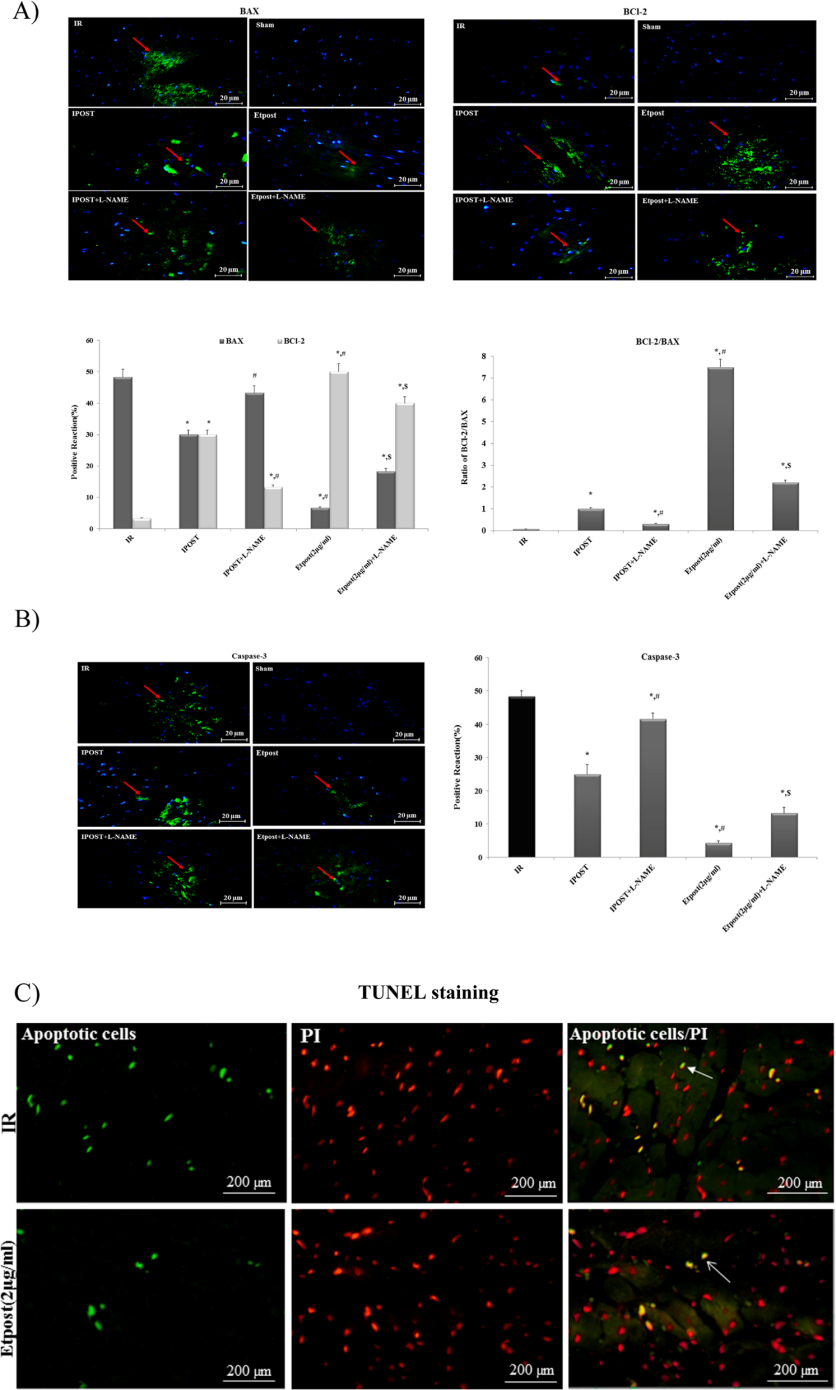
Anti-apoptosis effect of Etpost (2 μg/ml) on the isolated sections of rat heart tissue. Anti-apoptosis effect of ethyl acetate fraction from *P. reptans* on the isolated sections of rat heart tissue. The effect of IR, L-NAME, Etpost (2 μg/ml) and Etpost (2 μg/ml) + L-NAME on apoptotic markers. **A** Expression of BAX and BCl-2 proteins after nuclei staining with DAPI and obtained from fluorescence microscope (observed under 400 magnifications). **B** Expression of the levels of caspase-3 protein. **C** Representative TUNEL stained and captured by fluorescence microscopy of rat heart sections with IR and Etpost (2 μg/ml) groups. The green nuclear structures represent apoptotic cells. The nuclei are stained by PI. The data were expressed as mean ± SEM. *P < 0.05 vs. IR group. Etpost: ethyl acetate fraction of *P. reptans* root

Our results indicated that Etpost (2 μg/ml) significantly reduced caspase3 and BAX (pro-apoptotic protein) expression, while it remarkably increased BCl-2 (anti-apoptotic protein) against IR group. In addition, the administration of L-NAME abrogated the Etpost anti-apoptotic effect (Fig. 4A-B).

### TUNEL staining

Figure 4C shows TUNEL staining of Etpost (2 μg/ml) and IR on caspase 3 in isolated rat heart sections. It indicates that using Etpost (2 μg/ml) at the early phase of reperfusion caused a significant decrease in TUNEL-positive cardiomyocyte cells as regards to the IR group.

### Western blot analysis

**Fig. 5:**
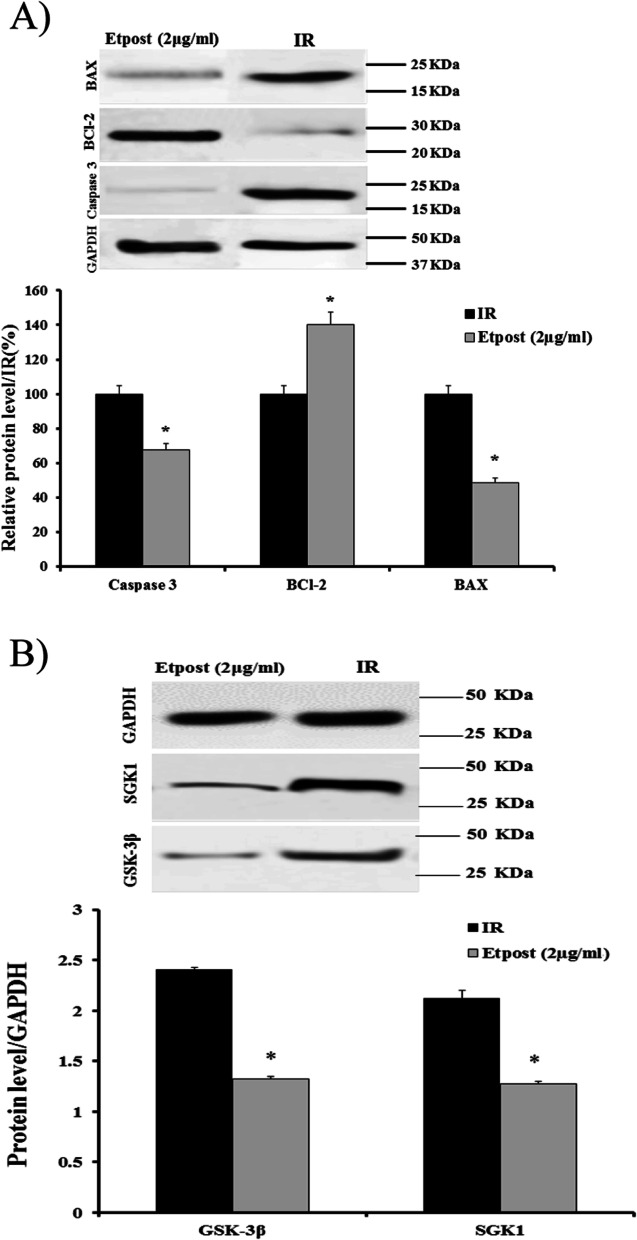
Western blot analysis. **A** Western blot analysis of BCl-2, BAX and levels of cleaved caspase-3 expression in heart tissue of Etpost (2 μg/ml) adjusted relative to GAPDH and IR. **B** Western blot analysis of GSK-3β and SGK1 expression in the heart tissue of Etpost (2 μg/ml) adjusted relative to GAPDH and IR. GAPDH was used as a loading control. The data were expressed as mean ± SEM. *P < 0.05 vs. IR group. Etpost: ethyl acetate fraction of *P. reptans* root

**Fig. 6:**
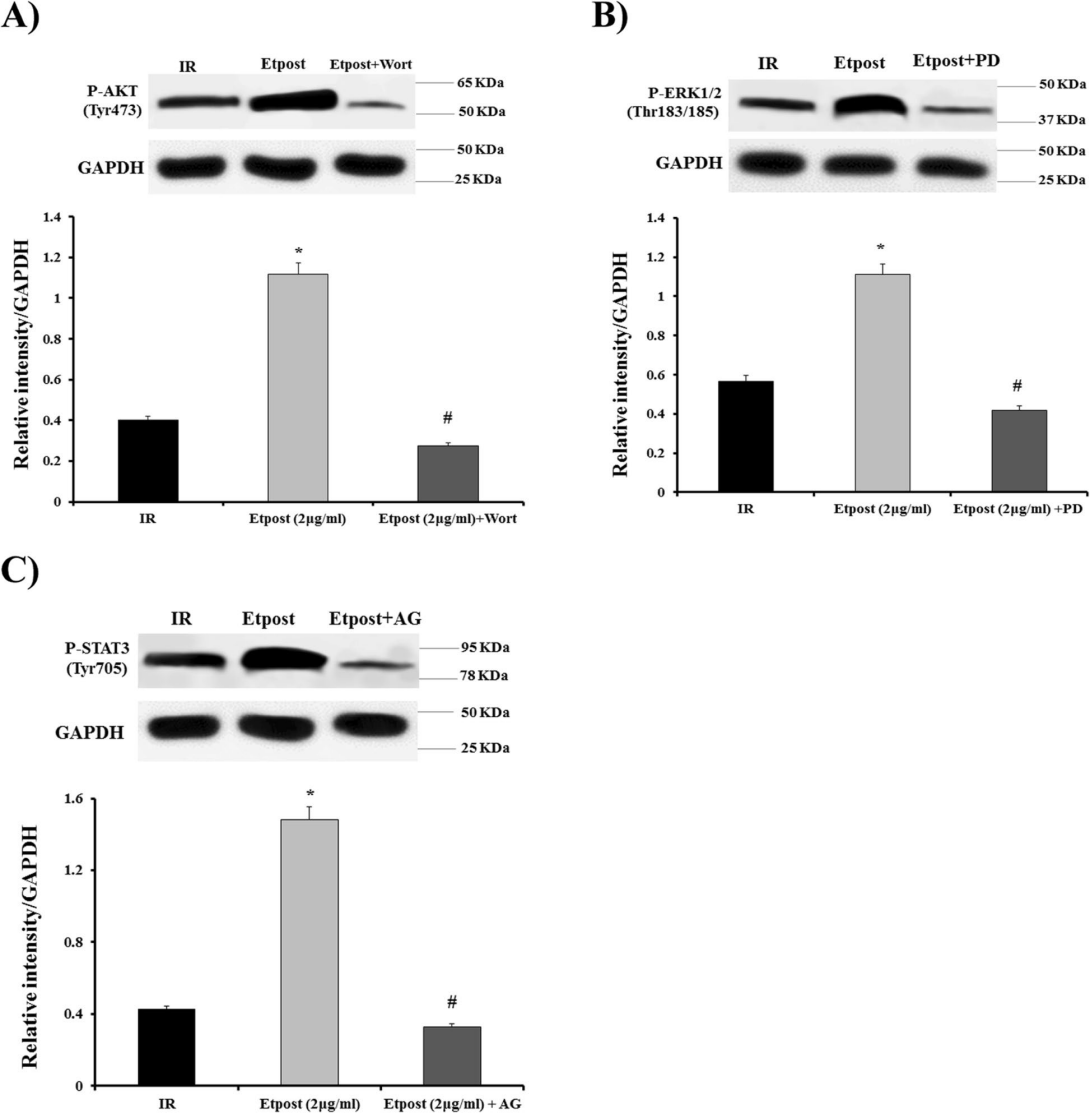
Western blot analysis. **A** Western blot analysis shows the relative intensity of AKT (Tyr473) phosphorylation in heart tissue of IR, Etpost (2 μg/ml) and Etpost+Wort groups that were adjusted relative to GAPDH. **B** Western blot analysis illustrates the relative intensity of ERK1/2 (Thr183/185) phosphorylation in heart tissue of IR, Etpost (2 μg/ml) and Etpost+PD groups that were adjusted relative to GAPDH. **C** Western blot analysis displays the relative intensity of STAT3 (Tyr705) phosphorylation in heart tissue of IR, Etpost (2 μg/ml) and Etpost+AG groups that were adjusted relative to GAPDH. The data were expressed as mean ± SEM. ^*^P < 0.05 vs. IR group and ^#^*P* < 0.05 vs. Etpost (2 μg/ml). Etpost: ethyl acetate fraction of *P. reptans* root; Wort: Wortmanin (PI3K/Akt inhibitor); PD: PD98059 (ERK inhibitor); AG: AG490 (JAK/STAT3 inhibitor)

From Fig. 5A and B, it can be seen that Etpost (2 μg/ml) could reduce the expression of apoptotic index, GSK-3β and SGK1 proteins during the early reperfusion phase in the IR model. Etpost (2 μg/ml) significantly decreased the expression of caspase 3, BAX, GSK-3β and SGK1 proteins by approximately 50% when compared with the IR group. As shown Fig. 6A-C, Etpost (2 μg/ml) significantly increased phosphorylation of AKT (Tyr473), ERK1/2 (Thr183/185) and STAT3 (Tyr705) regard to the IR group. Meanwhile, the administration of Wort, PD and AG490 with Etpost (2 μg/ml) decreased the AKT, ERK1/2 and STAT3 phosphorylation vs. Etpost (2 μg/ml) in a separate group.

### Hemodynamic properties

**Fig. 7:**
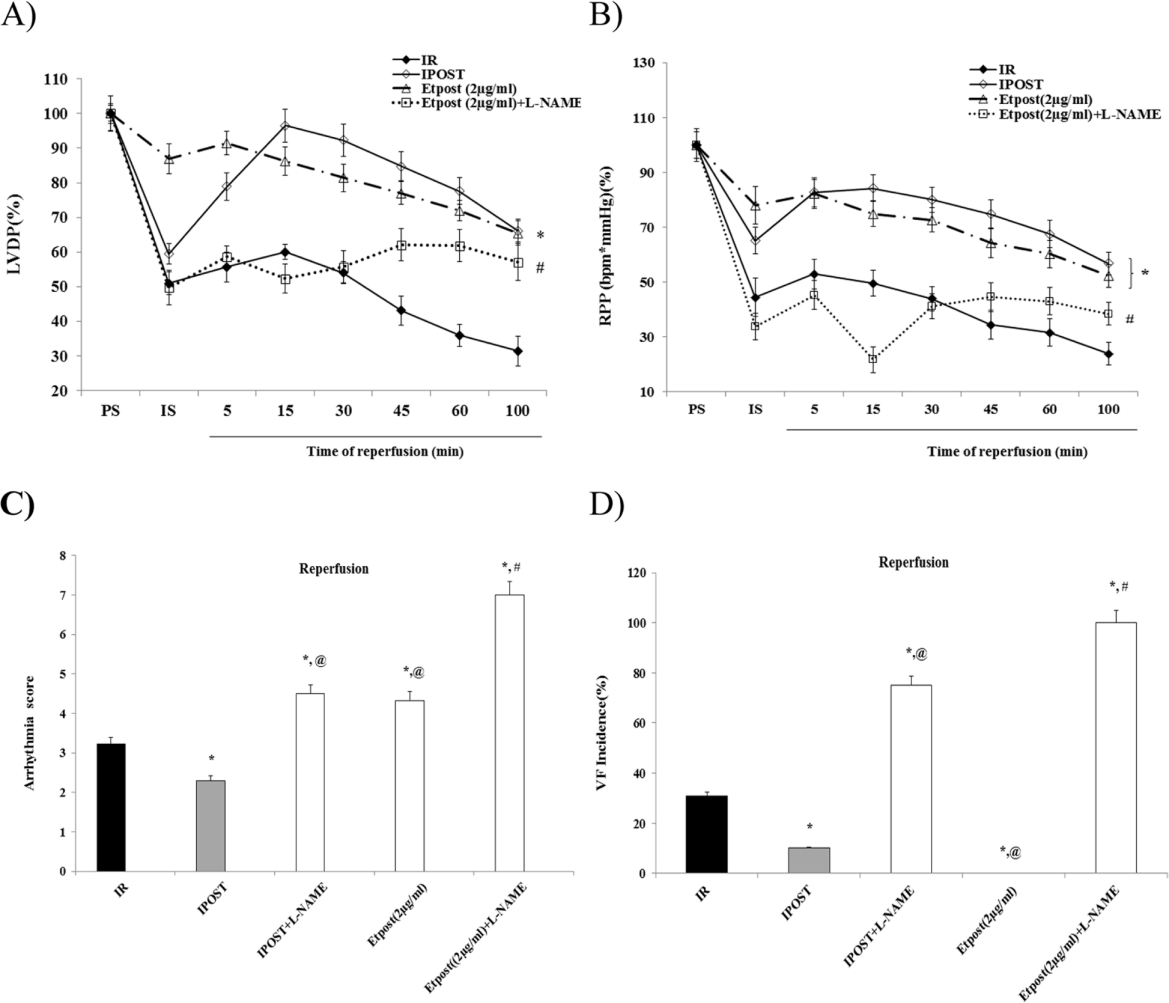
Effect of Etpost (2 μg/ml) on time course recovery of post-ischemic changes of hemodynamic parameters, Arrhythmia score and VF incidence in the isolated rat heart. **A** The effect of Etpost (2 μg/ml) and Etpost+L-NAME on LVDP, data are presented as % of per-ischemia (PS) values. ^*^ *P <* 0.05 vs. IR and ^#^ *P <* 0.05 vs. Etpost (2 μg/ml); **B** The effect of Etpost (2 μg/ml) and Etpost+L-NAME on Post-ischemic changes of rate pressure product (RPP) in the isolated rat heart. Data are presented as % of pre-ischemia (PS) values. ^*^ *P <* 0.05 vs. IR and ^#^ *P <* 0.05 vs. Etpost (2 μg/ml). **C** Arrhythmia scores calculation of the reperfusion period in the isolated rat heart. The effect of IR, IPOST, IPOST+ L-NAME, Etpost (2 μg/ml), and Etpost (2 μg/ml) + L-NAME on the arrhythmia score. Scale range is (0 to 7). ^*^ *P* < 0.05 vs. IR, ^@^ *P* < 0.05 vs. IPOST, and ^#^ *P* < 0.05 vs. Etpost; **D** The incidence of ventricular fibrillation (VF) calculation of the reperfusion period in the isolated rat heart. The effect of IR, IPOST, IPOST+ L-NAME, Etpost (2 μg/ml), and Etpost (2 μg/ml) + L-NAME on the incidence of VF. Data are presented as the percentage of incidence. ^*^ *P* < 0.05 vs. IR, ^@^ *P* < 0.05 vs. IPOST and ^#^ *P* < 0.05 vs. Etpost (2 μg/ml)

The effects of Etpost (2 μg/ml) on hemodynamic parameters are depicted in Table 2. Perfusion with Etpost (2 μg/ml) at the early reperfusion phase showed a significant recovery in RPP, HR, dP/dt max, and LVDP against the IR group during the reperfusion phase. In addition, applying L-NAME, AG, and Wort reduced the functional recovery effect of Etpost (2 μg/ml) on the hemodynamic parameters (Fig. 7A-B and Table 2), but PD and 5HD enhanced the beneficial hemodynamic effects of Etpost (2 μg/ml) during the reperfusion phase (Table 2).

### Assessment of arrhythmias

Despite, the increased arrhythmia score (4.33) by Etpost (2 μg/ml), it markedly reduced VF incidence (00.0%) against the IR (3.23, and 30.77%, respectively) group (Table 3). Additionally, except 5HD in the separate groups, perfusion of Wort, PD, AG, and L-NAME with Etpost (2 μg/ml) increased significantly, the arrhythmia score and VF incidence (*P* < 0.05, Fig. 7C-D & Table 3).

**Table 3:** Effect of Etpost (2 μg/ml) against the RISK/SAFE kinase inhibitors on arrhythmia score, VT, and VF incidence during the reperfusion phase of the IR model

	Arrhythmia score	VT incidence (%)	VF incidence (%)
IR (Con.)^@^	3.23 ± 0.85	69.23	30.77
IPOST (Con.)	2.30 ± 0.75^*^	50.00^*^	10.00^*^
Etpost (2 μg/ml)	4.33 ± 0.93^*^	100.0^*^	00.00^*^
Etpost (2 μg/ml) + Wort	6.50 ± 0.83^*,#^	100.0^*^	66.67^*,#^
Etpost (2 μg/ml) + PD	5.67 ± 0.34^*,#^	66.67^**#**^	100.0^*,#^
Etpost (2 μg/ml) + AG	6.25 ± 0.75^*,#^	50.0^*,#^	100.0^*,#^
Etpost (2 μg/ml) + HD	2.20 ± 0.17^*,#^	100.0^*^	00.000^*^
IPOST + Wort	7.00 ± 0.00^*,&^	100.0^*,&^	100.0^*,&^
IPOST + PD	7.00 ± 1.17^*,&^	100.0^*,&^	100.0^*,&^
IPOST + AG	5.75 ± 1.25^*,&^	50.00^*^	50.00^*,&^
IPOST + HD	3.00 ± 0.05^**&**^	33.33^*,&^	33.33^**&**^

*IR* ischemic reperfusion, *VT* ventricular tachycardia, *VF* ventricular fibrillation, *Etpost* Ethyl acetate fraction of *Potentilla reptans* root in postconditioning, *Wort* Wortmanin (PI3K/Akt inhibitor), *PD* PD98059 (ERK inhibitor), *AG* AG490 a JAK/STAT3 inhibitor, *HD* 5HD a mitoKATP channel blocker. The scale range of arrhythmia score is 0–7. Data are presented as mean ± SEM. *P < 0.05 vs. IR; ^&^ *P* < 0.05 vs. IPOST; ^#^ *P* < 0.05 vs. Etpost (2 μg/ml). @: [7]

## Discussion

In the current study, Etpost exerted anti-infarct, anti-stunning, and anti-arrhythmic effects as a postconditioning agent against IR injury. Our data proved the cardioprotective effect of Etpost, mediated via NO release, amplification of the *Nrf2* pathway, suppression of oxidant stress, activation of the RISK/SAFE pathways, and the reduction of the expression of GSK-3β and SGK1 proteins, which finally led to the activation of the anti-apoptotic pathways.

Owing to the multiple pharmacological effects of α-amyrin triterpenoids, they have aroused much interest in the treatment of cardiovascular disorders through increasing the activity of endogenous antioxidants, NO release, ROS balance, and decreasing the IS and MDA levels in IR injury [15]. A recent study revealed that Et contained polyphenols and exerted cardioprotective effects in isolated rat hearts [7]. Consistent with the present research, the cardioprotective properties of Etpost may be due to its active ingredients including triterpenes [8] and proanthocyanidins. In addition, other compounds might be involved in the biological effects of Etpost [11].

This study revealed the anti-infarct effect of Etpost, while it demonstrated that the application of L-NAME, Wort, PD, and AG490 reversed the beneficial effects of Etpost. However, the administration of 5HD did not cause any significant effect against the Etpost group. In our previous study, we demonstrated that the cardioprotective effect of Et is mediated via NO release [7]. Likewise, other studies have addressed the fact that several medicinal plants exert postconditioning effects via inciting NO release during IR [1, 2, 4]. Additionally, ROS balance during reperfusion and the activation of antioxidant enzymes, are thought to be mediated by NO release [7, 15]. Moreover, several studies have shown that the major cardioprotective signaling cascades involved in postconditioning agents for decreasing the reperfusion injury are, the salvage kinase, SAFE pathways, and the endothelial NO synthetase [1, 2, 4]. In line with the other studies, we showed that Etpost decreased the infarct size via NO release, activation of *Nrf2*, and ROS reduction. Therefore, the beneficial roles of Etpost are mediated via the RISK/SAFE signaling pathways during reperfusion.

However, reperfusion itself is associated with massive ROS burst during the initial phase, which plays a pivotal role in postconditioning [16]. Etpost significantly increased the expression of *Nrf2* during reperfusion, while the administration of L-NAME did not have any effect on the *Nrf2* expression. Moreover, Etpost showed a significant reduction in MDA levels and an increase in the activity of SOD and CAT. For the determination of its underlying mechanism, L-NAME was administered in combination with Etpost. A controversial result demonstrated that the depletion of NO did not reverse the antioxidant properties of Etpost. However, our results clearly showed that the beneficial protective effects of postconditioning are abolished by L-NAME in the IPOST+L-NAME group. This indicates the pivotal role of NO in enhancing the antioxidant capacity of cardiac tissue during reperfusion in the IR model. On the contrary, the inhibition of NOS did not reverse the protective effect of Etpost. To explain these findings, Picciano and Crane have confirmed that L-NAME has non-specific intrinsic activity [17]. Maybe these suggest the indirect/direct role of the complex signaling pathway of NO involved in the modulation of antioxidant activities of Etpost during reperfusion. Further studies are needed to assess the endogenous production of NO in the presence of Etpost to clearly demonstrate the fundamental role of NO in decreasing IS in the IR model.

It has been proposed that Etpost exerts its specific antioxidant activity, thereby activating the Nrf2 pathway, ROS scavenging, and stimulation of antioxidant activity. The result of the present study indicated the antioxidant properties of Etpost, mostly mediated via ROS scavenging and activating the *Nrf2* pathway during the reperfusion phase. In agreement with our findings, it has been described that Et exerts its antioxidant activities by boosting the Nrf2 pathway and possessing antioxidant components, such as polyphenolic and triterpenoids [7]. Triterpenoids and polyphenols attenuate the infarct size through their antioxidant effects, NO release, the *Nrf2* activation which suppresses oxidative stress, and decrease in MDA levels by increasing the coronary flow and anti-apoptotic effects [7, 15, 16, 18].

Furthermore, *P. reptans* exerts anti-apoptotic effects by decreasing IS and arrhythmias due to its antioxidant activities during the early phase of reperfusion. Etpost remarkably increased the anti-apoptotic index and decreased caspase-3 activity. Conversely, NO depletion abrogated the anti-apoptotic effect of Etpost. Likewise, the TUNEL assay and western blot confirmed the aforementioned findings. A growing body of evidence shows that polyphenols and triterpenoids which are the main active compounds of Etpost can induce their anti-apoptotic effects during reperfusion through NO release, antioxidant activities, the inhibition of lipoperoxidation, boosting the *Nrf2* expression, and activation of endogenous antioxidant enzymes [19–27]. Thus, Etpost depicted its intrinsic antioxidant and anti-apoptotic effects via NO release and ROS balance during reperfusion in the IR model.

To prove the downstream action mechanism of Etpost in the RISK /SAFE pathways, Wort, PD, AG490, and 5HD were perfused during the early phase of reperfusion. Our results demonstrated that the cardioprotective effect of Etpost was abolished after the aforementioned inhibitors except for 5HD. This confirmed that the RISK/SAFE pathways are involved in the cardioprotective and anti-apoptotic effects of Etpost during the early reperfusion phase. It is well-documented that the activation of RISK/SAFE pathways, by various plant secondary metabolites, can protect cells against apoptosis by decreasing the ratio of BAX/BCl-2 [2–4, 7, 28, 29]. Also, PI3K/Akt and GSK-3β are pivotal factors of postconditioning phenomena in IR injury, to prevent apoptosis and necrosis [28, 29]. Consistent with this, in the present study, the western blot analysis indicated that these two pathways were involved in the protective downstream signaling cascades of Etpost. Thus, Etpost exerts anti-infarct effects during the reperfusion phase through the downstream signaling pathways, including NO, PI3K/Akt, GSK-3β, and JAK/STAT3.

Furthermore, several studies indicated that the SAFE pathway, by activation of its downstream mediators, such as TNF-α and STAT-3 exerts cardioprotective and anti-apoptotic effects in both pre- or postconditioning [4, 30–32]. Besides, the data obtained from the analysis of molecular docking of Et compounds were in line with our pharmacological results, showing that they favorably blocked the GSK-3β protein. Especially, compound 1 is effectively stabilized in the binding pocket of the GSK-3β protein [8]. Therefore, the current research proved that the activation of the RISK/SAFE pathway is a fundamental step in the cardioprotective mechanism of Etpost.

The precise mechanism of Etpost in the improvement of the hemodynamic parameters and arrhythmia scores remains elusive. Etpost significantly enhanced the hemodynamic factors of the isolated rat heart via increasing LVDP, RPP, dp/dtmax, and coronary flow against the IR group. Additionally, Etpost showed an anti-arrhythmic effect through a decrease in arrhythmia scores and VF incidence. In the present experiment, Wort, PD, AG490, and L-NAME abolished the cardioprotective effects of Etpost, implying the contribution of NO, PI3K/Akt, GSK-3β, JAK/STAT3, and SGK1 pathways to anti-stunning and anti-arrhythmic effects during reperfusion. Transient opening of mitoKATP and massive production of ROS during reperfusion are associated with hemodynamic instability that occurs before the initiation of apoptosis and necrosis in IR [32].

Our previous data also demonstrated that the radical scavenging and antioxidant effects of *P. reptans* as a preconditioning agent could reduce ROS outburst and it could stabilize the hemodynamic parameters and exert anti-arrhythmic effects. These events may be indirectly mediated via the activation of NO release and the Nrf2 gene [7]. It is not clear whether the protective effects of Etpost are directly related to its anti-infarct properties, or indirectly from the intrinsic antioxidant effect of Etpost. Changes in the necrotic size of cardiomyocytes found in the infarct size, in an experimental model of hypoxia-induced myocardial injury, could affect the incidence of ventricular arrhythmias following reperfusion at the early phase [25]. Moreover, SGK1 has recently identified as a beneficial target for management of ventricular arrhythmia [33]. In addition, the inhibition of SGK1 reduces the action potential duration and prolongs repolarization. Therefore, ventricular arrhythmias can be reduced by abolishing elevated SGK1 in the presence of pathogenic causes [33, 34]. Our Western blot analysis and molecular docking showed that the active compounds of Etpost can inhibit SGK1 protein. Thus, the *P. reptans* root accomplishes its anti-stunning and anti-arrhythmic effects mostly either directly from decreasing IS or indirectly from its antioxidant and ROS scavenging effects.

Taken together, the present study sowed the anti-ischemic, anti-stunning, anti-arrhythmic, and anti-apoptotic effects of *P. reptans* root. These beneficial effects are due to the stimulation of antioxidant activity and NO release, as well as activating PI3K/Akt, JAK/STAT3, *Nrf2* pathways and inhibiting GSK-3β, SGK1, BAX, and caspase-3 during the early reperfusion phase. For to clarify the precise cardioprotective underlying mechanism of *P. reptans*, it is necessary further studies to confirm the cross talk between signaling pathways involved in reperfusion injury and purified isolated natural products of Et.

## Conclusion

In conclusion, Etpost reveals the cardioprotective and anti-apoptotic properties in the isolated rat hearts through NO release, amplification of the *Nrf2* pathway and endogenous antioxidant defense system, activation of RISK/SAFE pathways, and suppress of GSK-3β and SGK1 proteins, resulting in decreased oxidative stress and ROS. Our findings suggest *P. reptans* root as a potential postconditioning agent and RISK/SAFE pathway regulator. Because of multifactorial mechanism of reperfusion injury, Et can be a hopeful natural postconditioning agent for demoliting the pathogenesis of coronary artery disorders.

## Supplementary Information

**Additional file 1.**

## Data Availability

The datasets used and/or analysed during the current study are available from the corresponding author on reasonable request.

## Abbreviations

AAR: Area at risk
CAT: Catalase
Et: Ethyl acetate fraction of *Potentilla reptans* root
Etpost: Ethyl acetate fraction of *Potentilla reptans* root postconditioning
+dp/dtmax: Maximal rate of left ventricular systolic pressure
–dP/dtmax: Maximal rates of pressure fall
GSK-3β: Glycogen synthase kinase 3β
IPOST: Ischemic postconditioning
IR: Ischemia/reperfusion
IS: Infarct size
LAD: Ligating of the left anterior descending branch
L-NAME: Nω-nitro-L-arginine methyl ester (a nonspecific NOS inhibitor)
LVDP: Left ventricular diastolic pressure
LVEDP: Left ventricular end-diastolic pressure
LV: Left ventricular
MDA: Malondialdehyde
MI: Myocardial ischemia
NO: Nitric oxide
*P. reptans*: *Potentilla reptans*
ROS: Reactive oxygen species
RPP: Rate-pressure product
SGK1: Glucocorticoid regulated kinase-1
SOD: Superoxide anion dismutase
TTC: 2,3,5-triphenyl tetrazolium chloride
VF: Ventricular fibrillation
VT: Ventricular tachycardia
