# Supplementary material for: Potentilla reptans L. postconditioning protects reperfusion injury via the RISK/SAFE pathways in an isolated rat heart
Source: BMC Complement Med Ther. 2021 Nov 26;21:288. doi: 10.1186/s12906-021-03456-2 (PMC8620719; doi:10.1186/s12906-021-03456-2)

Original uncropped western-blot images

Figure 5 A. BAX protein after 100 min reperfusion. Western blot analysis was performed on the hearts of IR or rats treated with either Etpost (2 µg/ml) for early phase of reperfusion (15 min) according mentioned protocol in the manuscript. Etpost: ethyl acetate fraction of *P. reptans* root; IR: ischemia/reperfusion.

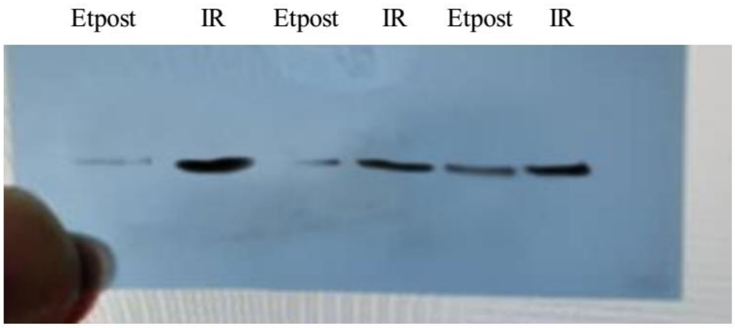

Figure 5 A. BCl-2 protein after 100 min reperfusion. Western blot analysis was performed on the hearts of IR or rats treated with either Etpost (2 µg/ml) for early phase of reperfusion (15 min) according mentioned protocol in the manuscript. Etpost: ethyl acetate fraction of *P. reptans* root; IR: ischemia/reperfusion.

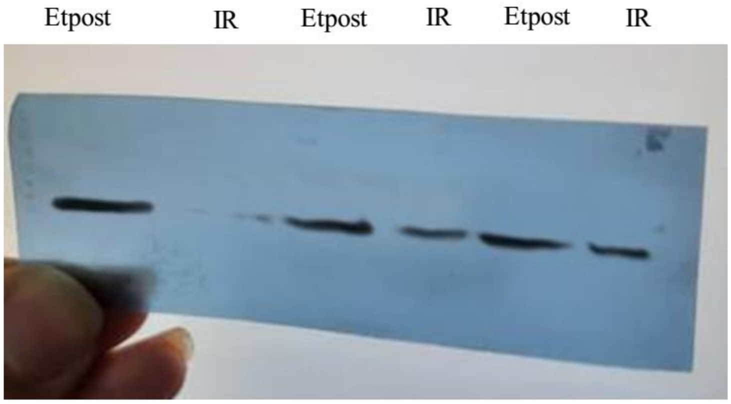

Figure 5 A. caspase-3 protein after 100 min reperfusion. Western blot analysis was performed on the hearts of IR or rats treated with either Etpost (2 µg/ml) for early phase of reperfusion (15 min) according mentioned protocol in the manuscript. Etpost: ethyl acetate fraction of *P. reptans* root; IR: ischemia/reperfusion.

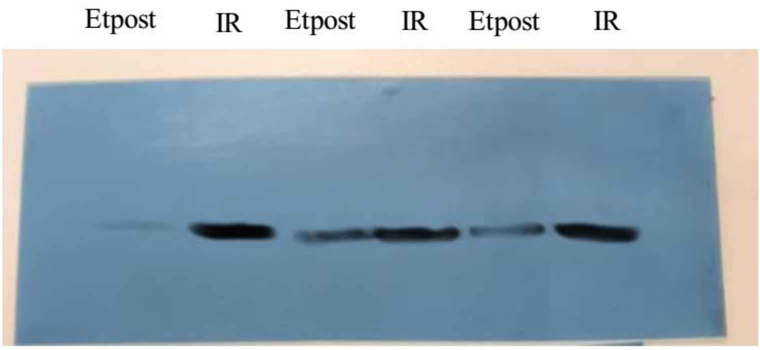

Figure 5 A. GAPDH protein after 100 min reperfusion. Western blot analysis was performed on the hearts of IR or rats treated with either Etpost (2 µg/ml) for early phase of reperfusion (15 min) according mentioned protocol in the manuscript. Etpost: ethyl acetate fraction of *P. reptans* root; IR: ischemia/reperfusion.

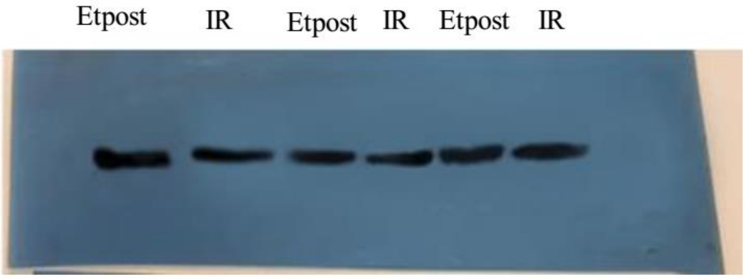

Figure 5 B. GSK-3β protein after 100 min reperfusion. Western blot analysis was performed on the hearts of IR or rats treated with either Etpost (2 µg/ml) for early phase of reperfusion (15 min) according mentioned protocol in the manuscript. Etpost: ethyl acetate fraction of *P. reptans* root; IR: ischemia/reperfusion.

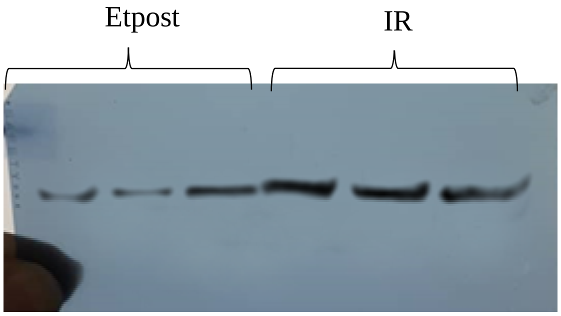

Figure 5 B. SGK1 protein after 100 min reperfusion. Western blot analysis was performed on the hearts of IR or rats treated with either Etpost (2 µg/ml) for early phase of reperfusion (15 min) according mentioned protocol in the manuscript. Etpost: ethyl acetate fraction of *P. reptans* root; IR: ischemia/reperfusion.

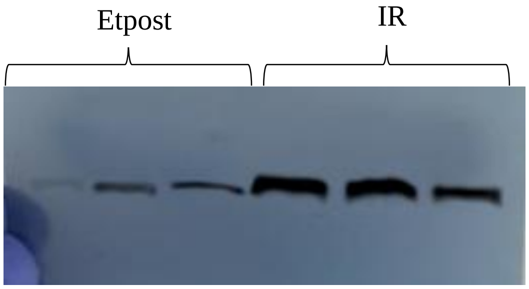

Figure 5 B. GAPDH protein after 100 min reperfusion. Western blot analysis was performed on the hearts of IR or rats treated with either Etpost (2 µg/ml) for early phase of reperfusion (15 min) according mentioned protocol in the manuscript. Etpost: ethyl acetate fraction of *P. reptans* root; IR: ischemia/reperfusion.

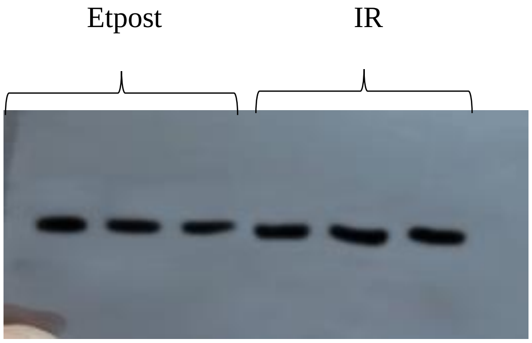

Figure 6 A. Phosphorylation of AKT protein (Tyr473) after 100 min reperfusion. Western blot analysis was performed on the hearts of IR or rats treated with either Etpost (2 µg/ml) and Etpost (2 µg/ml)+Wort for early phase of reperfusion (15 min) according mentioned protocol in the manuscript. Etpost: ethyl acetate fraction of *P. reptans* root; IR: ischemia/reperfusion; Wort: Wortmannin.

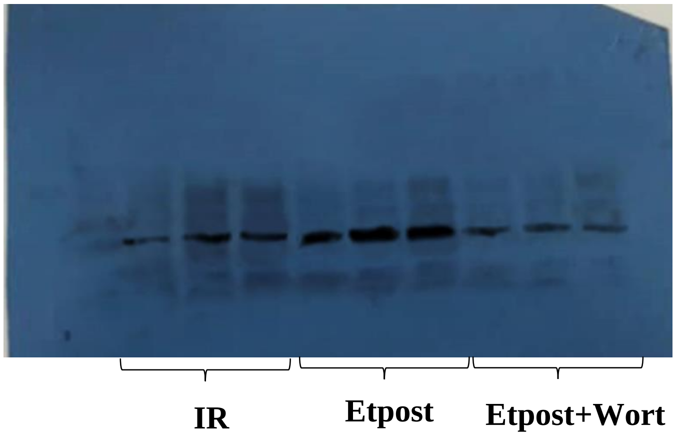

Figure 6 B. Phosphorylation of ERK1/2 protein (Thr183/185) after 100 min reperfusion. Western blot analysis was performed on the hearts of IR or rats treated with either Etpost (2 µg/ml) and Etpost (2 µg/ml)+PD for early phase of reperfusion (15 min) according mentioned protocol in the manuscript. Etpost: ethyl acetate fraction of *P. reptans* root; IR: ischemia/reperfusion; PD: PD98059 (2'-Amino-3'-methoxyflavone).

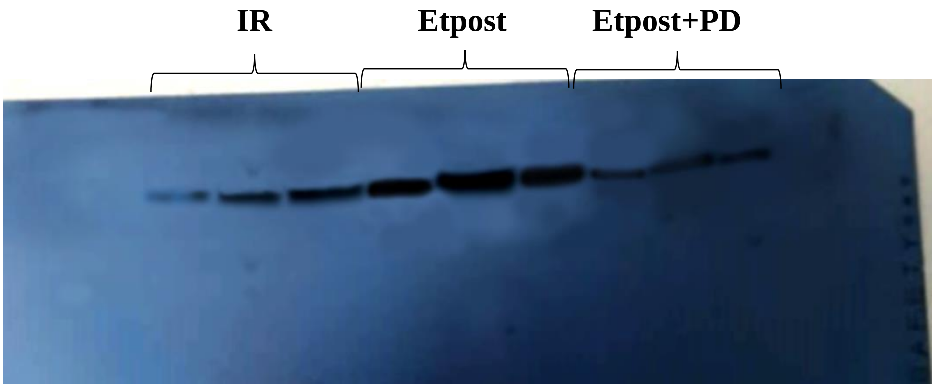

Figure 6 C. Phosphorylation of STAT3 protein (Tyr705) after 100 min reperfusion. Western blot analysis was performed on the hearts of IR or rats treated with either Etpost (2 µg/ml) and Etpost (2 µg/ml)+AG490 for early phase of reperfusion (15 min) according mentioned protocol in the manuscript. Etpost: ethyl acetate fraction of *P. reptans* root; IR: ischemia/reperfusion; AG: tyrphostin.

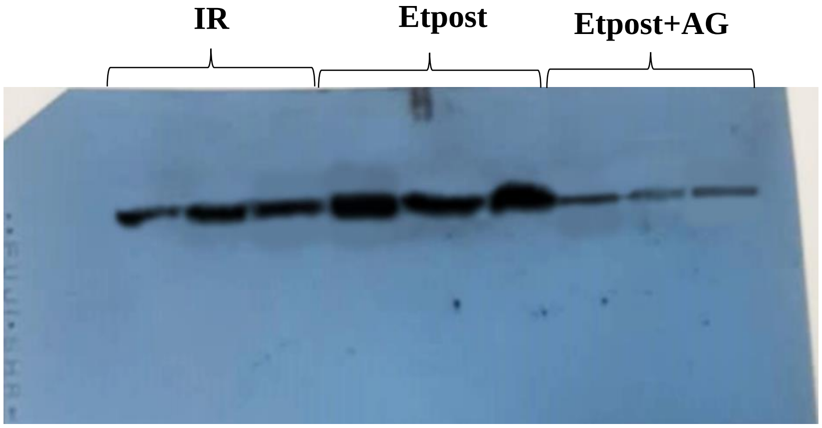

Figure 6. GAPDH protein after 100 min reperfusion. Western blot analysis was performed on the hearts of IR or rats treated with either Etpost (2 µg/ml) and Etpost (2 µg/ml)+inhibitors for early phase of reperfusion (15 min) according mentioned protocol in the manuscript. Etpost: ethyl acetate fraction of *P. reptans* root; IR: ischemia/reperfusion.

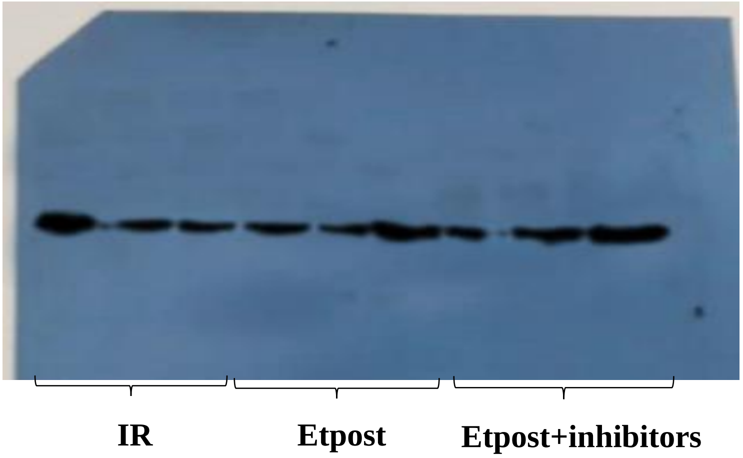

Supplement: Supplementary file 1 — Additional file 1. [file 12906_2021_3456_MOESM1_ESM.pdf]
